# Hospital and Institutional News

**Published:** 1914-07-11

**Authors:** 


### HOSPITAL AND INSTITUTIONAL NEWS.

### THEIR MAJESTIES AT GLASGOW ROYAL INFIRMARY.

During their long day at Glasgow on July 7 the King and Queen and Princess Mary saw many aspects of the city's life and activities. The first engagement, be it noted, on their crowded programme was the opening of the Diamond Jubilee Memorial block of the Royal Infirmary. Our leading article on p. 397 sums up the material facts concerning the planning and construction of this splendid building; but their Majesties' actual presence imparted great significance to the occasion in the eyes of the people of Glasgow. After the opening ceremony, their Majesties proceeded to inspect selected portions of the interior. One interesting feature of the morning was the presentation of Nurse Kate Bell, who was not only one of the infirmary staff in Lord Lister's time, but was actually closely associated with his work there for some years. The Electrical Institute also engaged their closest attention, and the taking of an electro-cardiographic tracing of a patient's heart was one of the first of a series of excellent demonstrations. The *x*-ray investigations were also of great merit. The movements of the stomach during digestion were examined (with the help of a bismuth meal), and the apparatus for inspecting the interior of the larynx and windpipe also created much interest.

### AT THE ROYAL HOSPITAL FOR SICK CHILDREN.

Later in the day a visit was paid to the Children's Hospital at Yorkhill, where, after the opening ceremony had been performed, the Queen unveiled the name-plate of the King George V. and Queen Mary Wards. Here, too, an inspection of the wards was made, and a cot allocated specially to children of the name of Brown attracted the King's attention. The operating theatre was also visited and was much admired. The growth of this splendid institution has been phenomenal, even for such a progressive city as Glasgow. As the address presented by the Duke of Montrose reminded their Majesties, the hospital was started with thirty-two cots in 1882; an out-patient department was added in 1888, and twenty-four more cots (outside the city) in 1903. Now two hundred patients will be admitted to the new hospital when it is finished, and the plans admit of further expansion to accommodate three hundred. Last of all, their Majesties paid a flying visit to the Western Infirmary. The institutional section of the Glasgow citizens was thus very fully recognised, for in spite of the numerous claims of the civic and mercantile and university communities, their Majesties actually visited three hospitals, at two of which important ceremonies were undertaken.

### THE CONSUMPTION CONFERENCE AT LEEDS.

The sixth annual conference of the National Association for the Prevention of Consumption and other Forms of Tuberculosis was held at Leeds on July 7 and 8. On the opening day the morning session was occupied by a discussion of the house in relation to tuberculosis, in which several of the highest authorities on this disease took part. One and all agreed upon the effects of bad housing conditions in fostering the spread of consumption. The urgency of condemning back-to-back houses and of abolishing slums was a theme which occupied several of the speakers; while others laid stress in addition on the gospel of personal hygiene and of avoidance of alcohol and similar factors in the spread of tuberculosis. Dr. T. D. Lister, whose utterances on this question are always forceful, emphasised also the need for enforcing proper notification of tuberculosis, and the importance of Local Government Board recognition of tuberculosis as an infective disease.

### SURGICAL TUBERCULOSIS IN CHILDHOOD.

On the afternoon of July 7 the subject of the hour was "Tuberculosis, especially Surgical, in Childhood," and the high standard of the morning discussion was well maintained. Mr. Lawford Knaggs emphasised the beneficial results of very radical removal of tuberculous cervical glands, results which many physicians do not yet appreciate. He placated the latter, however, by declaring that tuberculosis is always medical in the sense that the control of the tuberculous patient should rest with the physician, not the surgeon. Mr. S. W. Daw eulogised the tuberculosis hospital, situated in the country, as a necessary annexe of the big urban general hospital for the treatment of non-phthisical tuberculosis. Alderman Shelmerdine read a paper, which excited great interest, on the electrical sterilisation of milk, which he claims to have demonstrated as practicable and commercially feasible. The second day of the conference, July 8, was wholly given up to the consideration of domiciliary measures against tuberculosis. The most picturesque paper was that of Dr. Woodcock, consulting tuberculosis officer for Leeds; but there was a plentiful supply of other good contributions. The prominent feature of several of these was the earnestness of the pleas for after-care, lest the good work of the sanatorium be undone by unfavourable influences and relapse ensue.

### THE ROYAL SALOP INFIRMARY.

His Majesty has graciously given permission that the County Hospital at Shrewsbury shall be in future known as the Royal Salop Infirmary, in commemoration of his visit to Shrewsbury on Friday of last week. This infirmary is quite one of the most venerable of provincial hospitals, for its foundation dates back to 1745. At the annual meeting of the Salop Infirmary, presided over by Mr. Beville Stainer, M.P., the resignation was announced of Mr. J. Jenks, the Secretary for twenty years past, on grounds of health. High tributes were paid by several speakers to Mr. Jenks's devotion to the institution, and a resolution was passed accepting the resignation with deep regret. The report for last year's work shows many satisfactory features: increases of income under most heads, and a fine record of work done. One of the most satisfactory features of the accounts is the reduction of the adverse balance from £2,891 at the beginning of the year to £1,676 at the end of it. It is true that this result is obtained by including legacies amounting to £3,148 in the summary of income, of which only £1,499 appears on the expense side as invested; so that without the legacies the institution would have made a loss of £434 instead of a profit. But as institutional finances go, the Salop Infirmary has a good record. It is particularly pleasing to note that church and chapel donations amount to £862; it is evident that in Shrewsbury the clergy recognise their responsibilities in this matter very clearly and definitely.

### THE NEW SANATORIUM AT HULL.

When the King lately visited Hull he laid the foundation stone of a new sanatorium, under the Corporation, on the Cottingham Castle Estate, some five miles from the city. The cost of the projected sanatorium will be about £20,000, and towards this capital expenditure the Government will pay three-fifths up to and not exceeding £90 per bed. The Insurance Committee will also pay to the Health Committee of the Corporation of Hull an agreed sum per bed for the patients whom they send in. The accommodation will include a block of forty beds for males, divided into one ward of ten beds and fifteen wards of two beds each; and a block of twenty beds for females, divided into ten wards of two beds each; in each case with all the necessary offices, recreation-rooms, and so forth. Attached to the women's ward there will be a common dining-room and sculleries. It will be noticed that at £90 a bed this design hardly accounts for £20,000. The explanation appears to be that another sixty beds are being provided on the estate for other purposes (fever cases). The administrative block, it would seem, is to be common to both the sanatorium and the other wards. Mr. J. H. Hirst, city architect, is the designer of the buildings.

### HOSPITAL EXPANSION IN BIRKENHEAD.

The committee of the Birkenhead Borough Hospital have come to the conclusion that the institution needs to be enlarged to meet the growing demands on its services and to be made generally efficient. They have, therefore, issued an appeal for £15,000 as the least sum with which they can carry out the necessary alterations. Founded in 1828 as a dispensary, the hospital function dates from 1845, and the present building from 1862. In 1901 a nurses' home and a new women's ward were added, and other alterations have raised the capacity of the hospital to seventy-six beds and three cots (the small number of the latter is accounted for by the fact that Birkenhead supports a separate children's hospital). As the income of the charity at the present time is between £4,000 and £5,000, the raising of £15,000 for capital expenditure is an ambitious proposal, but not one which Birkenhead need hesitate to support and to carry through. According to the local press, this hospital up to a few years ago was not managed in a way that enlisted the confidence of the public; but latterly, says this same critic, a great improvement has occurred, the hospital has become a very important institution in the borough, and its enlargement is a public necessity.

### THE BIRMINGHAM CONCERTS COMMITTEE.

The Lord Mayor of Birmingham received at the Council House a few days ago the members of the Birmingham and District Sunday Evening Concerts Committee, and was handed a cheque for £500 by Mr. G. A. Parker, the concert secretary. Of this sum, by desire of the committee, £450 goes to the Women's Hospital at Sparkhill, and the rest to Dr. Taylor's Memorial Home. Mr. Parker stated that during the seven years of the Sunday concerts more than £2,500 has been handed over to the Women's Hospital, the authorities of which have now allotted the committee two beds. This is a fine record, and the Lord Mayor struck the right note in acknowledging the debt of gratitude owing to those who organise these concerts. He emphasised also the value of the work being done at Sparkhill, especially in regard to after-care. The Honorary Treasurer (Mr. H. Martin) of the Women's Hospital, to whom the Lord Mayor then handed the cheque, gratefully thanked the committee for their donation, and stated that the number of beds has this year been increased to ninety-four. He also said there never was a time when money was more needed by the hospital.

### THE ROYAL ALBERT INFIRMARY, WIGAN.

The report of the board of management, presented to the annual meeting of the subscribers on June 22, discloses a fine record of work done and of courage in tackling work waiting to be done. In spite of an increase in ordinary income of nearly £600, and in total income of £1,600, the debt on maintenance account has risen by £1,070 to £5,547. The building fund debt is, however, substantially less than it was a year ago. Out of an ordinary income of £11,821, the workpeople's contributions amount to £9,136, a magnificent total, which proves that self-help is fully understood and practised in Wigan. It is stated that one or two large legacies are likely to be received shortly, and it has been decided to venture upon certain further improvements which will be somewhat costly. Open-air balconies and improved sanitation for the children's wards are to be provided; and, more important still, the new out-patient scheme is to be put in hand. The late Mr. S. Wood, when Mayor, set out to raise £25,000 as a King Edward Memorial, of which £10,000 was to be ear-marked for the purpose of this improvement. Whether or not the death of the prime mover of the scheme affected its success, at any rate only £3,700 is available. With this, however, the Board feel justified in going on, and the new department will probably be started within three months. It will be seen that the management are not afraid to spend large sums on needed improvements, even though their institution is not free from debt incurred on existing buildings. They have been so magnificently supported by the workpeople in regard to maintenance that it may be hoped some of the wealthy inhabitants of Lancashire will come forward and assist in relieving the heavy burdens on the capital account entailed by the progressive policy of the infirmary.

### A LEPER HOSPITAL IN ESSEX.

The late Lord Strathcona bequeathed £5,000 for the establishment of a hospital for lepers in England, and it is stated that a farm has been acquired for this purpose near Binacre, four miles from Chelmsford. Plans have been prepared for a building, but meanwhile the existing farmhouse will be utilised for the accommodation of those in need of immediate treatment. There are supposed to be about twenty lepers in England at the present time, but it is not stated how many of them are thought likely to apply for admission to this lazarette. The scheme includes, besides those actually suffering from leprosy, patients with other disfiguring maladies. Twelve beds will be provided at the outset, and others will be added as required, and as financial considerations permit. The scheme is in charge of the St. Giles' Association for the Treatment of Incurable Diseases of the Skin, and the patients will be ministered to by the Brotherhood of the Divine Compassion. London specialists have expressed their willingness to co-operate in a consultative capacity.

### MORE MEDICAL ARCHÆOLOGY.

Charing Cross Hospital is fortunate just now in the literary activities of its physicians. A few days ago Dr. Hunter's projected history was published; and now Dr. Galloway, the senior physician, has issued a little monograph on the associations of the site and neighbourhood of the hospital, the proceeds of the sale of which he is devoting to the hospital. "Historical Sketches of Old Charing" deals mainly with the history of the Convent of St. Mary Roncevall at Charing Cross, dissolved 1544, and with Eleanor of Castile and the crosses erected to her memory at Charing and elsewhere. Dr. Galloway's researches are of much interest to all London lovers, not merely to the *alumni* of his hospital; they are reprinted from the *Charing Cross Hospital Gazette*. Yet another piece of London archæology by a medical author is the historical poem by Major Greenwood, M.D., entitled "London," with notes, map, and plan.

### THE NEW APPOINTMENTS AT ST. GEORGE'S HOSPITAL.

When a short time ago the matron (Miss McCall Anderson) and the assistant-matron (Miss Watson) of St. George's Hospital simultaneously resigned, the institutional world was excusably speculative as to the reasons for this "double event," and curious as to their successors. The latter question has now been set at rest by the appointment of Miss Elsie Cooper, assistant-matron of the Royal Free Hospital, to be matron at St. George's; and of Miss Babtie to be assistant-matron under her. Both these selections appear to be excellent, and to give promise that the high administrative standard set by the outgoing office-holders will be fully maintained. Miss Babtie is a sister of Surgeon-General Babtie, V.C., now in high command in India. She was trained at St. George's, where she was also ward sister and night superintendent, before her appointment a year or two ago as assistant-matron at the Royal Sea Bathing Hospital.

### THE SECRETARYSHIP OF ST. MARK'S HOSPITAL, CITY ROAD.

An advertisement has appeared in a medical paper asking for applications for the post of secretary to St. Mark's Hospital for Cancer, Fistula, and other Diseases of the Rectum, City Road. Inquiries which we have made elicit no information as to the manner in which the vacancy has arisen beyond the statement that Mr. A. W. Sowden, the late secretary, is no longer attending to discharge the duties of the post. It is a little difficult to understand why so much mystery should be created about so ordinary a matter; it can only be assumed that the authorities have their own reasons for avoiding publicity. The obvious disadvantage of their plan is that the greater part of the institutional world will probably fail to get news of the vacancy, so that the area of choice of a new secretary may be unduly restricted in consequence. The somewhat unusual course has also been taken of not disclosing in the advertisement the emoluments attaching to the post.

### THE DEVONSHIRE HOSPITAL AT BUXTON.

At the annual meeting of the subscribers to the Devonshire Hospital and Buxton Bath Charity, held at the hospital on June 26, a gratifying record of steady progress was disclosed. Both the number of patients treated and the income of the institution were the largest on record during the fifty-five years of the hospital's existence. The chairman, Sir E. T. D. Cotton-Jodrell, K.C.B., while congratulating the subscribers on the satisfactory condition of the hospital and on the splendid work which it performs, took occasion to plead for increased support from the churches. Collections from ten places of worship were allotted to the hospital during the year, and amounted to £82. We agree with Sir E. T. D. Cotton-Jodrell that the hospital has a legitimate claim for help from many more churches in the neighbourhood, from amongst whose congregations large numbers of the patients treated are derived; and we feel sure that his suggestions for appealing to those congregations for help have only to be made widely known to be promptly responded to. The total number of beds available for the in-patients has now been raised to 316. The building committee are contriving to carry out several important structural improvements without diminishing the capacity of the hospital. These include the erection of new baths, the renewal of the whole of the heating apparatus of the hospital, new boilers, rearrangement of the water supply, and the making of a subway. The chairman also referred to the research work being carried on in the hospital, and to the Duke of Devonshire's appeal for £25,000 for this purpose.

### THE BRISTOL GENERAL HOSPITAL.

It may interest many readers to know that the new wing of this hospital is to be officially opened on the 27th instant, but that it cannot be brought into full work until a later date. The workmen are now putting the last touches. The laundry and other additions to this important hospital are all in working order. Considerable interest attaches to these extensions owing to the high repute which the management of this institution has deservedly maintained for many years.

### PROPOSED COTTAGE HOSPITAL AT MALTBY.

A mass meeting of miners at Maltby was held lately to consider a proposal concerning the establishment of a cottage hospital there. It transpired that Lord and Lady Scarbrough are anxious to build and equip such an institution in memory of the late Lieutenant R. A. Ashton, the son of Lady Scarbrough. The condition imposed is that the upkeep and maintenance of the hospital shall be discharged by the public of Maltby. The estimated annual sum required is £750, and the colliery employees have offered to guarantee one-third of this sum. The directors of the colliery company offered to provide one quarter of the annual cost, after deducting subscriptions from the general public. The letter containing this offer was ordered to lie on the table, which means (we presume) that for the present it is neither accepted nor rejected. It was the general opinion of the meeting that a contribution of one penny per week per man would provide the necessary funds; but the miners' representatives objected to pledge themselves to this, on the ground that, as the colliery developed, almost the whole cost of maintenance might be thus defrayed. This attitude seems to overlook the fact that as the colliery develops the need for a cottage hospital will grow more and more acute, and that by the time the miners grow so numerous they and their dependants will form of necessity the vast majority of those admitted to the institution.

### UNDERGRADUATES AND VOLUNTARY HOSPITALS.

An auxiliary fund in support of the Royal Hospital for Diseases of the Chest, City Road, E.C., has been started at various Oxford Colleges. A meeting to inaugurate the fund and to interest Oxford men in the fight against tuberculosis was addressed by Mr. Stuart de la Rue, chairman of the hospital, and by Dr. Barty King, Dean of the Medical School; and as a result an effective organisation has been started. This organisation will, it is hoped, assist the hospital in its efforts to equip itself as a teaching and research centre of the first rank, and will also, it is hoped, interest Oxford men, as they go down, in their lay responsibilities in connection with hospital work. The idea is certainly novel and praiseworthy. It remains to be seen whether any great accession of funds results from this new departure; but in any case the diffusion amongst undergraduates of a knowledge of the tuberculosis problem, and of the needs of voluntary hospitals in order that they may play a worthy part in its solution, can be productive of nothing but good. We congratulate the management of the Royal Hospital for Diseases of the Chest on their bright idea, and on their clear perception of the paramount value of personal service if voluntary institutions are to maintain their prosperity and to serve the community worthily.

### AN INSTITUTIONAL PILGRIMAGE.

Some thirteen years ago, as the result of a bet, a crew of four members of the resident medical staff of the Queen's Hospital, Birmingham, undertook to row on the canal from Birmingham to Worcester in less than twelve hours. The conditions of the trip were that the hospital "barge" should be used, an old and heavy double-sculling boat; that all the fifty-eight locks on the course should be worked by two of the rowers; and that no special sculls should be used in the various tunnels, which are too narrow for full-sized oars, but the boat got through by a pair of sculls on one side working against the rudder. Since the first of these curious pilgrimages several other hospital crews have attempted the same feat. The record time of eight hours fifty-five minutes was established in 1906, but this year the residents' crew has lowered these figures to eight hours thirteen minutes; nine or ten hours appears to be about the usual time taken. One of the tunnels, near King's Norton, is two miles in length, and must prove a heart-breaking section of the journey when negotiated according to the conditions laid down. Athletic performances and hospital residence do not as a rule go well together; so great credit attaches to the four doctors who have this year set up a new record, and it is to be hoped that it will be regularly attacked in future years.

### SANATORIA AND AFTER-CARE.

Those responsible for modern sanatoria treatment, especially since the passing of the Insurance Act, have become especially alive to the importance of after-care work. In the case of the Kelling Sanatorium efforts have been made to discuss with patients the nature of the work which awaits them on their discharge. In many cases, the committee state, employers have promised to re-engage discharged patients, and have often altered the conditions under which the work has to be resumed. Sometimes these alterations have made the work the most desirable thing available. In the case of those patients who have no work in view, the after-care committee advises which kind of occupation is within their powers, and they are recommended to various agencies or friends. Any kind of work which can be taught at the sanatorium during the period of treatment, like gardening or poultry farming, is inculcated, and for early cases in which the disease has been arrested emigration is sometimes advised. The value of this after-care work is felt, however, to be diminished by the absence of after-care committees under the control of the County and Borough Councils, to whom leaving cases could be referred. The hope is also expressed that provision for after-care will find a place in every permanent scheme that may be undertaken, and the after-care committee expresses the belief that such work is doubly important, not only to prevent relapses, but to counteract "the excessive, unwarranted fear of the consumptive" felt by employers. It cannot be doubted that as the causes of disease and of relapses are pushed further back the futility of any treatment unco-ordinated by preventive and after-care measures will be more strongly felt, and so long as the institutions do not attempt to take more responsibility upon them than they themselves are able to cope with, the comprehensive attempts to place after-care on a wide basis, as a further link in the chain of the "campaign" against tuberculosis, are certainly to be welcomed.

### COLOSSAL ENDOWMENTS IN AMERICA.

The scale of private munificence in the United States long ago outstripped anything to which we can point in this country; but even in that land of dollars no institution, we fancy, can ever have received so large a single gift as that which Mr. Henry Ford, the motor-car manufacturer, a fortnight ago bestowed upon the Detroit General Hospital. The sum in question is variously stated at £600,000 and £1,000,000, and Mr. Ford has stated that he intends to convert the hospital into an institution for the study and prevention of tuberculosis and cancer. Almost simultaneously it is announced that Mr. Rockefeller is giving £510,000 for medical research in general. There is, of course, no institutional philanthropist in Great Britain whose bounty can be matched with that of these American millionaires; but it is timely to recall that Sir James Caird, who has just presented Sir E. Shackleton with £24,000 to complete the equipment of his Polar expedition, has been a generous patron of several hospitals in Scotland. In 1897 he founded the Dundee Maternity Hospital with a gift of £6,000, and he gave £24,000 soon after for the Cancer Hospital in the same city. A sanatorium for consumption cost him £10,000, and among his other large benefactions may be especially mentioned those connected with the out-patient department of the Dundee Royal Infirmary and the Home of Rest. He also presented the British Association for the Advancement of Science with £10,000 on one occasion.

### A FLOURISHING CONCERN.

The twenty-third annual report of the Nurses' Co-operation shows a record of continued progress in all respects, and demonstrates the advantages to everyone concerned of the first-rate business management which such a very large co-operation renders possible. The number of nurses on the staff is now 524, and no fewer than 6,715 cases were nursed in 1913. The finance section is perhaps the most instructive part of the whole report. It shows that the gross receipts from patients reached the huge sum of £53,654, of which £50,489 was paid to the nurses who earned it. The difference between these two sums, £3,165, together with interest on investments, constitutes the income of the society. This income is dealt with in paying all the working expenses of the business, together with the charge in respect of the nurses' home and flats; it is satisfactory to know that there is a balance on the year's working of £344. Roughly speaking, this large and successful co-operation is "run" at a cost of only 6 per cent. upon the receipts, the remaining 94 per cent. being paid to the members of the staff. This result reflects great credit on all concerned in the management, which consists chiefly of St. Thomas's men (or their wives). Incidentally, we may note that the Finance Committee consists of three men and six women, four of the latter being representatives of the nurses.

### JUDGE MELLOR ON A BAD PRACTICE.

The institutional world is well aware of the existence of a serious abuse in connection with accident cases admitted to hospital—namely, the visits of the touts of shady solicitors whose object is to persuade injured persons to employ their principals in compensation proceedings, usually on the half-profit system. That this system flourishes, partly by connivance of bribed hospital employees, is common knowledge. A different and probably rarer abuse was disclosed in the Manchester County Court the other day, when Judge Mellor made some caustic comments on the conduct of an employer in visiting one of his injured workmen in a hospital only four hours after the latter had broken his thigh, and in persuading him to sign an agreement accepting compensation for a limited period and admitting that the employer was not liable. We fully agree with the Judge's remarks, and, apart altogether from the abuse of the institution which he denounced, it may be added that few men are in a fit state to transact legal business within four hours of suffering from the injury which this patient had.

### THE COUNCIL ELECTION OF THE ROYAL COLLEGE OF SURGEONS.

In a recent issue (The Hospital, June 20, p. 334) allusion was made to the exceptional interest taken in this year's Council election of the English Royal College of Surgeons; and the proof of that interest is supplied by the record poll that has resulted. Mr. Thorburn, Manchester, headed it with 534 votes; Mr. Ballance, the only member seeking re-election, was second with 434; Mr. Eccles polled 351, Mr. Boyd 337, and Mr. Ryall 304. Three candidates, Messrs. Lawford, Openshaw, and Yearsley, had been recommended by the Society of Members as supporters of the movement for admitting the members to a share in the government of the College. Although none of the three secured election, two of them did very well and were not far off. Thus, our forecast that no supporter of this policy was likely to be elected has been borne out by the polling figures. In any event, the new members of Council, as will be evident to those who have studied their individual records, cannot fail to bring to their work on the Council genuine ability of varied types, and also the prestige of reputation and position worthily earned in their past careers.

### NIGHTINGALE SCHOLARSHIPS.

The Nightingale Fund and the use it was put to by Miss Nightingale in founding the school of nursing attached to St. Thomas's Hospital are, of course, more than fifty years old. The Council of the Fund are considering an extension of its work with the object of providing some training in the administrative and the sociological sides of nursing beyond that in nursing pure and simple. The call for women with these higher qualifications grows ever more insistent, while at the same time the constantly increasing curriculum of the modern nurses' training leaves her less and less time in which to cultivate them. A tentative scheme is being evolved to supply the omission. A certain number of scholarships will be offered every year to those who have completed a full three-years' training at a recognised school. These scholarships will be tenable for a year at the Household and Social Science Department of King's College for Women, in buildings now being erected at Campden Hill. Here special facilities for practical work are being provided: laboratories, kitchens, laundries are part of the scheme. A residential hostel for those who desire it is also part of the buildings. The training will, it is stated, be thoroughly practical, and the syllabus will be at first somewhat tentative, so that there will be no difficulty in adopting such alterations as experience may dictate.

### THE WAR OFFICE COMMITTEE ON VOLUNTARY AID DETACHMENTS.

As recorded in our last issue (p. 371), the working and organisation of the Voluntary Aid Detachments of the Territorial Force are to be reviewed by a committee. In a letter to the *Times* Mrs. M. A. Stobart protests against the exclusively male *personnel* of this War Office Committee. She points out that in London there are 1,327 women enrolled in Voluntary Aid Detachments, as against 211 men. "If," she writes, "women are incapable of taking a share in the organisation of work which is pre-eminently woman's work, they are incapable of responsibility in a national crisis. . . . But if, as I maintain, women are very capable of participating in the organisation of work concerned with the sick and wounded, then the exclusion of the female sex from this committee of inquiry is an insult to all women and a special grievance to those thousands of women who have . . . sacrificed much money, time, and energy in the cause of national defence." It will be admitted that Mrs. Stobart presents a strong case. Those who read our comments on her book on Red Cross work in the Balkans ("War and Women," reviewed in The Hospital, February 7, 1914, p. 502) will remember that we do not see eye to eye with her on every point. It gives us the more pleasure to state that on this occasion she has our entire sympathy in her protest, and there is no one from whom such a protest could more fittingly proceed.

### DEATHS BY POISONING.

The annual report of the Registrar-General of Births, Deaths, and Marriages, which has just been issued, records the usual heavy death-rate from poisons, and bears evidence that there is no diminution of deaths from this cause. Over a thousand fatal cases are reported, but 280 are due to anæsthetics, and can hardly be classified as ordinary poisoning cases. In 220 cases death was due to poisons accidentally administered, and in 545 to poisons self-administered by suicides. The report deals with the year 1912, and shows that during that period no less than forty-eight deaths were caused by the accidental administration of opium preparations or derivatives either as an overdose or by mistake for some other substance; carbolic acid caused seventeen fatal accidents, veronal nine, and trional three. Opium preparations were also used by fifty-two suicides, but the three poisonous substances most often used by suicides were carbolic acid, hydrochloric acid, and oxalic acid, which together account for 258 cases. The classification of deaths under anæsthetics which was introduced in the last report has been adhered to, and returns are published, as a process of secondary classification, of all deaths on the certificates relating to which any mention of the administration of an anæsthetic is made.

### THIS WEEK'S DRUG MARKET.

There has been little, if any, improvement in business during the week, but the drug market is not altogether without interest. The expected advance in the price of santonin has been announced, the value now being well over twenty times the price at which this drug could be freely bought at one time; there have been rumours that the Russian Government may not renew the monopoly in wormseed (from which santonin is produced), which is now held by a syndicate whose factories are in Turkestan, but the rumours await confirmation. Should they be confirmed, which seems doubtful, the future of the market would be very uncertain. Quicksilver is lower in value, and it is not altogether improbable that quotations for corrosive sublimate, calomel, and other salts of mercury may be reduced. The value of opium tends upwards, and morphine is rather dearer. A further advance in the price of citric acid is probable. Tartaric acid also has an upward tendency, and the same may be written of cream of tartar. It is not unlikely that buchu leaves will advance in price. The value of olive oil is well maintained. Carbolic acid is inclined towards higher prices.

